# Half-Life of Serum Elimination of Perfluorooctanesulfonate,Perfluorohexanesulfonate, and Perfluorooctanoate in Retired Fluorochemical Production Workers

**DOI:** 10.1289/ehp.10009

**Published:** 2007-06-12

**Authors:** Geary W. Olsen, Jean M. Burris, David J. Ehresman, John W. Froehlich, Andrew M. Seacat, John L. Butenhoff, Larry R. Zobel

**Affiliations:** 1 Medical Department, 3M Company, St. Paul, Minnesota, USA; 2 Pace Analytical Laboratory, St. Paul, Minnesota, USA

**Keywords:** biomonitoring, perfluoroalkyl acids, perfluorohexanesulfonate, perfluorooctanesulfonate, perfluorooctanoate, PFHS, PFOA, PFOS, pharmacokinetics

## Abstract

**Background:**

The presence of perfluorooctanesulfonate (PFOS), perfluorohexanesulfonate (PFHS), and perfluorooctanoate (PFOA) has been reported in humans and wildlife. Pharmacokinetic differences have been observed in laboratory animals.

**Objective:**

The purpose of this observational study was to estimate the elimination half-life of PFOS, PFHS, and PFOA from human serum.

**Methods:**

Twenty-six (24 male, 2 female) retired fluorochemical production workers, with no additional occupational exposure, had periodic blood samples collected over 5 years, with serum stored in plastic vials at −80°C. At the end of the study, we used HPLC-mass spectrometry to analyze the samples, with quantification based on the ion ratios for PFOS and PFHS and the internal standard ^18^O_2_-PFOS. For PFOA, quantitation was based on the internal standard ^13^C_2_-PFOA.

**Results:**

The arithmetic mean initial serum concentrations were as follows: PFOS, 799 ng/mL (range, 145–3,490); PFHS, 290 ng/mL (range, 16–1,295); and PFOA, 691 ng/mL (range, 72–5,100). For each of the 26 subjects, the elimination appeared linear on a semi-log plot of concentration versus time; therefore, we used a first-order model for estimation. The arithmetic and geometric mean half-lives of serum elimination, respectively, were 5.4 years [95% confidence interval (CI), 3.9–6.9] and 4.8 years (95% CI, 4.0–5.8) for PFOS; 8.5 years (95% CI, 6.4–10.6) and 7.3 years (95% CI, 5.8–9.2) for PFHS; and 3.8 years (95% CI, 3.1–4.4) and 3.5 years (95% CI, 3.0–4.1) for PFOA.

**Conclusions:**

Based on these data, humans appear to have a long half-life of serum elimination of PFOS, PFHS, and PFOA. Differences in species-specific pharmacokinetics may be due, in part, to a saturable renal resorption process.

Perfluorooctanesulfonate [PFOS; CF_3_(CF_2_)_7_SO_3_^−^] and its acid salts were derived from perfluorooctanesulfonyl fluoride [POSF; CF_3_(CF_2_)_7_SO_2_F]. Major product applications were developed using POSF through formation of N-alkylsulfonamides that were used in surfactants, paper and packaging treatments, and surface protectants (e.g., carpet, upholstery, textiles). Depending on the specific functional derivitization or polymerization, these POSF-based products may have degraded or metabolized, to an undetermined degree, to PFOS, a stable and persistent end product that has a widespread presence in the general population (Butenhoff et al. 2006) and wildlife (Houde et al. 2006).

Salts of perfluorooctanoic acid, in particular ammonium perfluorooctanoate (APFO), have been used as surfactants and processing aids in the production of fluoropolymers and fluoro-elastomers. Industrial production of the salts of perfluorooctanoic acid occur through electrochemical fluorination and telomerization. Perfluorooctanoate [PFOA; CF_3_(CF_2_)_6_COO^−^], the dissociated carboxylate anion, has been measured in humans worldwide but generally at lower nanogram per milliliter concentrations than PFOS (Houde et al. 2006).

In rats, PFOS and PFOA are not metabolized and enter into the enterohepatic circulation (Johnson et al. 1984; Kemper 2003; Kuslikis et al. 1992; Vanden Heuvel et al. 1991). Because of the stability of the carbon–fluorine bond and the high electronegativity of perfluorinated alkyl acids, metabolism would not be favored; thus, perfluorohexanesulfonate (PFHS) is also not expected to be metabolized.

Based on the determination of volumes of distribution from single-dose intravenous studies in cynomolgus monkeys, the distributions of PFOS, PFHS, and PFOA are primarily extracellular (Butenhoff et al. 2004; Noker and Gorman 2003a, 2003b). Kerstner-Wood et al. (2003) found PFOS, PFHS, and PFOA to be highly bound in rat, monkey, and human plasma over a concentration range of 1–500 μg/mL. When incubated with human plasma protein fractions, all three compounds were highly bound (99.7 to > 99.9%) to albumin, and showed affinity for β-lipoproteins (95.6, 64.1, and 39.6% for PFOS, PFHS, and PFOA, respectively). Some binding to α - and γ -globulin fractions and minor interactions with transferrin (PFHS and PFOA) were also noted. PFOS and PFOA have been shown to compete for fatty acid binding sites on liver fatty acid binding protein, with PFOS giving the stronger response (Luebker et al. 2002).

The elimination rates of PFOS and PFHS have been studied in male and female cynomolgus monkeys after intravenous dosing (Noker and Gorman 2003a, 2003b) and for PFOS after repeated oral dosing (Seacat et al. 2002). Noker and Gorman (2003a, 2003b) reported mean (± SD) terminal elimination half-lives, ranging from 88 to 146 days (132 ± 13 days for males and 110 ± 26 days for females) for PFOS and 49 to 200 days (141 ± 52 days for males and 87 ± 47 days for females) for PFHS, after intravenous dosing of three male and three female cynomolgus monkeys in separate experiments, with no significant difference between males and females or between the two compounds. Seacat et al. (2002) reported an approximate terminal elimination half-life of 200 days for PFOS in male and female cynomolgus monkeys during 1 year immediately following 6 months of daily oral dosing with either 0.15 or 0.75 mg/kg PFOS.

Elimination rates in species other than the monkey have been determined for PFOS and PFOA. Within 89 days after a single intravenous dose of ^14^C-PFOS, 30% of the ^14^C was excreted in the urine and 12% in the feces of male rats (Johnson et al. 1979). For PFOA, significant interspecies differences have been observed (Hundley et al. 2006; Kudo and Kawashima 2003), and differential expression of organic anion transporters in renal proximal tubule cells have been suggested as an explanation for sex differences in the rat (Kudo et al. 2002) and low elimination rates in humans (Andersen et al. 2006).

The purpose of the present study was to estimate the serum elimination half-life of PFOS, PFHS, and PFOA in humans through the long-term follow-up of retired fluoro-chemical production workers. Although these retirees were no longer occupationally exposed, their serum concentrations were expected to be considerably higher than those of the general population.

## Materials and Methods

### Study population

Retirees from the 3M Company, Decatur, Alabama, facility were eligible for the study if they had retired between January 1995 and onset of the study in November 1998. The retirees were invited to participate based on having prior work assignments in fluorochemical production. Thirty-four individuals were identified and 24 (22 males, 2 females) agreed to participate (71%). In addition, 3 retirees from the 3M, Cottage Grove, Minnesota, chemical division were also directly invited to participate. Their primary exposure would have occurred in the APFO production area. The study was approved by the 3M Institutional Review Board. All study participants gave informed consent before study initiation. Participation was voluntary and subjects could withdraw from the study at any time, although none did. Participants received $50 per blood collection.

Blood collection (approximately 10 mL/ collection) for the 24 Decatur retirees began in November 1998, with subsequent collections for each employee in June and November 1999, May 2000, February 2001, January 2002, January 2003, and March 2004. Blood collections almost always occurred on the same day for all of the retirees. Maximum follow-up was 1,945 days (5.3 years). Blood collection for the 3 Cottage Grove participants began in June 1999 and ended in March 2004, with five additional blood collections interspersed between these dates. For these 3 retirees, blood collections were generally not on the same day but within a short (1–2 weeks) period of time. Maximum follow-up was 1,744 days (4.8 years).

Serum was stored in plastic vials at −80°C. All samples for PFOA analysis were analyzed in batches of 30 samples/day at the end of the study. Samples for PFHS and PFOS were analyzed separately from PFOA samples.

### Laboratory analysis

#### Chemicals

Potassium salt of PFOS, potassium salt of PFHS, and ammonium salt of PFOA were obtained from internal sources at the 3M Company (St. Paul, MN). The internal standard for PFOS and PFHS analyses was a labeled perfluorooctane-sulfonate (^18^O_2_-PFOS) obtained from the Research Triangle Institute (Research Triangle Park, NC). For PFOA, we used a labeled ^13^C_2_-PFOA (DuPont de Nemours & Co., Wilmington, DE) as the internal standard. The acetonitrile and methanol used for extraction purposes were “Distilled in Glass” grade solvents from Burdick and Jackson (Morris Township, NJ). Reagent grade formic acid, reagent grade potassium hydroxide, ammonium acetate, and ammonium sulfate were obtained from J.T. Baker (Phillipsburg, NJ). The ammonium sulfate was washed with methanol and dried before use. All other chemicals and solvents were used without further purification.

#### Standard solutions and quality control materials

We adjusted the initial weights of the primary standards for impurities and the salts present such that the concentrations in methanol represented the negative ion concentrations as measured in the serum matrix analyzed. Stock standard solutions were prepared in methanol at concentrations of 0.1 mg/mL; separate stock standards were prepared (0.1 mg/mL) in methanol for spiking control materials; and additional standard dilutions were completed in methanol as required for spiking into the standard curve matrices. Rabbit or calf serum controls were spiked at target concentrations in bulk to cover the range of the assay as completed. The quality control solutions were placed on a magnetic stirrer overnight to mix thoroughly, and aliquots were frozen at −80°C until use. Blank rabbit or calf serum was evaluated with each run, and the spiked matrix was used for matrix-matched standard curve extractions.

Both intrabatch and interbatch run accuracy and precision measurements were performed. For PFOA, we used three spiked samples with targeted means of 80, 240, and 750 ng/mL. Recovered means ranged between 93.3 and 101.5%. Coefficients of variation (CVs) ranged between 1.4 and 3.8. For PFOS and PFHS, we used 80 and 350 ng/mL targeted mean spiked samples. For PFOS, recovered means ranged between 91.0 and 99.7%, with CVs between 2.2 and 4.9. For PFHS, recovered means ranged between 88.6 and 105.2%, with CVs ranging between 6.9 and 11.9.

The lower limit of quantitation (LOQ) was set as the lowest acceptable standard value fitted on the standard curves used. For all analyses, regardless of instrumentation used, the lower limit of quantitation was 10.0 ng/mL. PFOA was analyzed using the Finnigan TSQ 7000 instrument (Thermo Electron Corp., Waltham, MA) using the parent negative ions (Q1 MS) only. Based on a signal-to-noise ratio of 5:1, the lower limit of detection for PFOA was 1.6 ng/mL. PFOS and PFHS were analyzed using the API 4000 instrument (Applied Biosystems, Foster, CA) used in the tandem mass spectrometry (MS-MS) mode. Based on a signal-to-noise ratio of 5:1, the lower limit of detection was 1.0 ng/mL for PFOS and was 0.5 ng/mL for PFHS.

#### Sample extraction

Initial extractions were completed at an acidic pH coupled with an alkaline back extraction technique. All primary extraction tubes were new, disposable polypropylene tubes to which an appropriate amount of internal standard was added before the initial extraction. The initial extraction was based on 250 μL serum, 300 μL 1.0 N formic acid, and 300 μL saturated ammonium sulfate added together in the primary extraction tube. The tubes were then briefly mixed by vortexing for 30 sec. The primary extraction solvent was acetonitrile (5 mL). After the addition of the acetonitrile, all tubes were shaken on a mechanical shaker for 30 min. The primary extraction tubes were removed from the shaker and centrifuged at 2,500 × *g.* The top layer (acetonitrile) containing the compounds of interest (analytes and internal standard) was decanted to a clean, labeled tube and the acetonitrile was dried down to residual aqueous using the LabConCo evaporator (LabConCo, Kansas City, MO).

#### Back extraction

Residual aqueous from the primary extraction was further diluted with 300 μL deionized water and made alkaline with the addition of 300 μL of 1.0 N potassium hydroxide solution. This mixture was then vortexed and re-extracted (alkaline back extraction) using 7.0 mL methyl *tert*-butyl ether (MTBE) on a mechanical shaker for 20 min. After centrifugation at 2,500 × *g* for 5 min, the top layer (MTBE) was transferred to a clean polypropylene tube for solvent evaporation using a gentle stream of nitrogen gas in a temperature-controlled water bath (N-EVAP; Organomation Associates Inc., Berlin, MA).

The tubes were promptly removed after the MTBE solvent had evaporated to dryness. Samples were then reconstituted using 400 μL of solution containing 2 mM ammonium acetate (50%) and acetonitrile (50%). After vortexing, the mobile phase mixture was transferred to polypropylene microliter inserts. These inserts were then placed in a 2-mL glass microvial, capped, and placed on either the API 4000 liquid chromatography-MS-MS system or the TSQ 7000 for analysis.

#### HPLC conditions

Both the TSQ 7000 and the API 4000 instruments were equipped with identical high-performance liquid chromatography (HPLC) columns. We used MacMod (Chadds Ford, PA) ACE C-18 columns (100 × 2.1 mm i.d. columns, 5-μm particle size). A 3-μm guard column was used in front of the analytical column (10 × 2.1 mm i.d.). The TSQ 7000 flow rates were optimized for an isocratic separation of the branched chain isomers for the PFOA analysis at approximately 0.25 mL/min. The mobile phase composition was approximately 50% acetonitrile and 50% 2 mM ammonium acetate. A 5-μL injection was employed to introduce the sample to the TSQ 7000 mass spectrometer.

The liquid chromatograph used with the API 4000 system was the Agilent 1100 series HPLC system (Santa Clara, CA). The API 4000 instrument was operated with a flow rate of 0.35 mL/min, using a mobile phase mixture similar to that used in the analysis of PFOA as described above.

#### LC-MS analysis

PFOA analysis was completed using a TSQ-7000 mass spectrometer operating in Q1 (parent ion) mode. PFOS and PFHS analyses were completed simultaneously using an API 4000 mass spectrometer operating in multiple reaction–monitoring (product ion) mode. PFOS and PFHS extracts were reevaluated for all samples using the TSQ-7000 (parent ion) for both validations of the new instrument and to justify choosing the product ion at 80 amu for quantitation.

PFOS and PFHS each form product ions at 80 and 99 amu, and both product ions were monitored during this study. Quantitation was based on the area ratio between the 80-amu product ion formed by the analytes and the internal standard (^18^O_2_-PFOS) production formed at 84 amu. Quantitation of the PFOS using the TSQ 7000 was completed on the 499-amu ion for PFOS, the 399-amu ion formed for PFHS, and the 503-amu ion formed for the ^18^O_2_-PFOS internal standard ion. PFOA was completed based on the formation of the electrospray negative ions formed at 413 amu and the negative ion formed from ^13^C_2_PFOA at 415 amu.

The TSQ 7000 system was operated in the electrospray ionization mode using negative ionization detection with a constant source potential of 3.0 kV applied. The following masses were monitored for peak intensities [base peak (Q1 mass *m/z*)]: PFOA (413.0); PFOA internal standard, C_6_F_15_^13^C_2_OO^−^ (415.0); PFOS (499.0); PFOS internal standard, C_8_F_17_S^18^O_2_ O^−^ (503.0); and PFHS (399.0).

The TSQ 7000 capillary inlet heater was held at a constant 300°C. PFOA quantitative calculations were based on the ion ratios between PFOA and the added internal standard (C_6_F_15_^13^C_2_OO^−^). PFOS and PFHS quantitative calculations were based on the ion ratios between the compounds of interest (PFOS, PFHS) and the added internal standard (C_8_F_17_S^18^O_2_O^−^). Linear regression analysis was then cmpleted with standards weighted at 1/*x*. Concentration was plotted along the *x*-axis and the peak area ratio was plotted on the *y*-axis.

The API 4000 system was operated in the negative ion mode using TurboIon spray operation. All API 4000 source parameters were optimized according to the manufacturer’s guidelines. The specific ions monitored were base peak Q1 (mass *m/z*); Q3 mass (product ions *m/z*): PFOS (Q1, 499.0; Q3, 80 and 99); PFOS internal standard (Q1, 503.0; Q3, 84 and 103); and PFHS (Q1, 399.0; Q3, 80 and 99).

#### LC-MS instrument data analysis

Data acquisition and analysis were completed for all blanks, standards, quality control samples, and unknowns using Analyst software on the API 4000 or the Excalibur software package on the TSQ 7000. Manual integration of isomer peaks was completed only where necessary to include the area under the branched isomers present as part of the total peak area integrated. No branched PFOA isomer peak was measurable when compared with the low standard at 10 ng/mL. However, using a lower detection limit resulted in branched PFOA concentrations of approximately 1% (range, 0.1–6.0%). The PFOA standard contained the linear isomer at 78% and the combined branched chain isomers at 22%. Linear regression calibration curves weighted by the reciprocal of the standard amount (1/*x*) were used for quantitation.

### Data analysis

During the review of the subjects’ questionnaire data, we determined that three Decatur retirees likely had additional occupational exposure after their initial blood collection in November 1998. One retiree had intermittently worked with fluorochemicals at the Decatur facility throughout the study period. This retiree was excluded from the entire data analysis. A second Decatur retiree had intermittently worked in the Decatur facility until February 2001; therefore, his data were truncated until that point in time. A third Decatur retiree likely had occupational exposure to PFOA between his initial (November 1998) and second (June 1999) blood collections that affected his assessment; therefore, we used June 1999 as his initial time point for measurement of PFOA. All other subjects had all of their data analyzed.

The 26 retirees had 197 blood collections included for half-life analyses. A total of 189 (96%) of the 197 blood collections had sufficient sample available for split analyses. Spearman Rho correlations exceeded 0.98 for each analyte.

We used WinNonlin software, version 4.1 (Pharsight Corporation, Mountain View, CA) to calculate the half-life of elimination based on the mean value of the split analyses. The data were linear when plotted as the logarithm of serum concentration versus time. Therefore, we assumed a first order model (Medinsky and Klaassen 1996).

We used multiple regression to determine statistically significant (*p* < 0.05) associations between the half-life of elimination for PFOS, PFHS, and PFOA and explanatory variables including initial and end-of-study perfluorochemical concentration, age at study onset, years worked, and years since retirement.

## Results

Table 1 presents the demographic information of the 26 subjects by ascending order of their initial serum PFOS concentration as shown in Table 2. At the time of the initial blood collection, the mean age of the 26 subjects was 61 years (range, 55–75 years). These subjects had worked a mean of 31 years (range, 20–36 years), and they had been retired on average 2.6 years (range, 0.4–11.5 years). Based on their work history records, their lifetime usual jobs at either the 3M Decatur or Cottage Grove facility were categorized as electrochemical fluorination cell operators (*n* = 3), chemical operators (*n* = 6), maintenance workers (*n* = 5), foremen (*n* = 6), laboratory technicians (*n* = 3), and other (*n* = 2: warehouseman and engineer). Their mean length of study follow-up was 1,849 days (range, 1,139–1,945 days) equivalent to a mean of 5.0 years (range, 3.1–5.3 years). Two of the retired subjects died during the study follow-up period, which limited each of their length of follow-up to 1,524 days (4.2 years).

Based on the individual data presented in Table 2, the arithmetic mean initial serum concentrations were as follows: PFOS, 799 ng/mL (median, 626 ng/mL; range, 145–3,490 ng/mL); PFHS, 290 ng/mL (median, 193 ng/mL; range, 16–1,295 ng/mL); and PFOA, 691 ng/mL (median, 408 ng/mL; range, 72–5,100 ng/mL). The arithmetic mean end-of-study serum concentrations were PFOS, 403 ng/mL (median 295 ng/mL; range, 37–1,740 ng/mL); PFHS, 182 ng/mL (median 117 ng/mL; range 10–791 ng/mL); and PFOA, 262 ng/mL (median 148 ng/mL; range 17–2,435 ng/mL).

**Figure 1:**
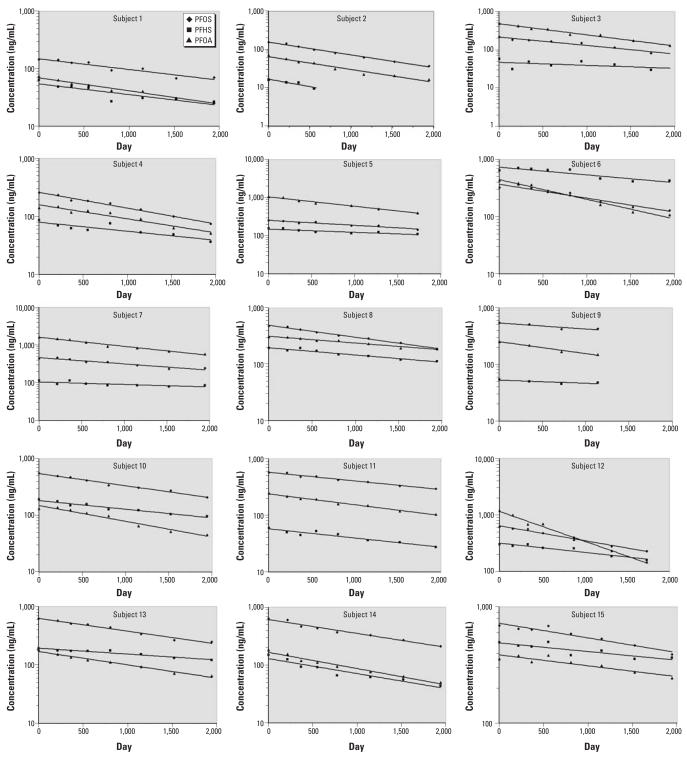

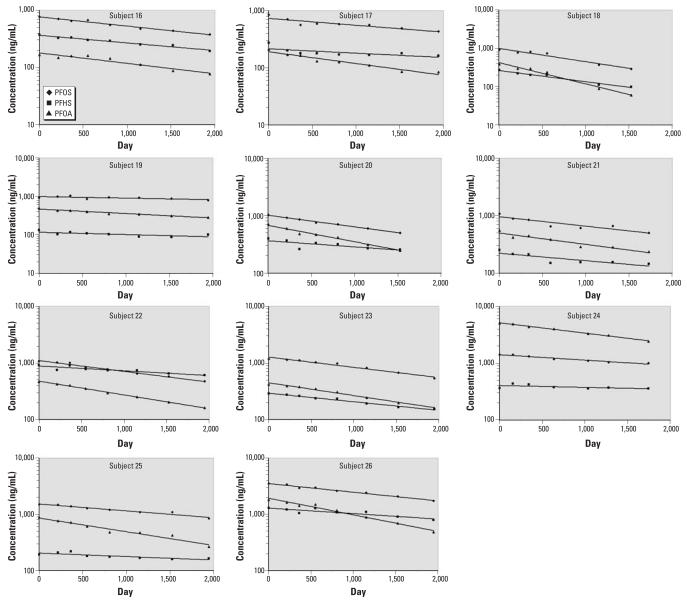
Semi-log plots of PFOS, PFHS, and PFOA concentrations by time (days) for subjects 1–26. Data points indicate observed values and the line shows predicted value.

We examined semi-log plot graphs of concentration by time for each of the 26 subjects (Figure 1). Individual serum elimination half-lives for PFOS, PFHS, and PFOA using a first order model are presented in Table 2. Assuming a log-normal distribution, Pearson correlation coefficients for the log half-lives (days) of serum elimination were *r* = 0.72 (*p* < 0.0001) for PFOS and PFOA; *r* = 0.65 (*p* = 0.0003) for PFOS and PFHS; and *r* = 0.46 (*p* = 0.02) for PFOA and PFHS.

**Figure 2:**
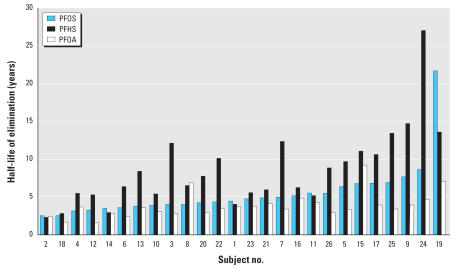
Half-life (years) of serum elimination for PFOS, PFHS, and PFOA (in ascending order for PFOS) for 26 retired fluorochemical production workers by subject number.

Table 3 shows arithmetic and geometric means and their respective 95% confidence intervals (CIs) for the serum elimination half-lives of PFOS, PFHS, and PFOA. The arithmetic mean half-lives of elimination for PFOS, PFHS, and PFOA were 5.4 years (95% CI, 3.9–6.9), 8.5 years (95% CI, 6.4–10.6), and 3.8 years (95% CI, 3.1–4.4), respectively. Median values and ranges are also reported in Table 3 and indicate the right skewness of the distribution, as visualized in Figure 2. Excluding subjects 19 and 24, the two subjects with the highest half-lives of serum elimination for PFOS and PFHS that appear in Figure 2, the arithmetic mean half-lives of elimination for PFOS and PFHS reduced to 4.8 years (95% CI, 4.1–5.4) and 7.8 years (95% CI, 6.3–9.3), respectively. These arithmetic mean values were then similar to the geometric mean and median values reported in Table 3 when each of these subjects was included in the analyses. The two female subjects (subjects 7 and 25) had arithmetic mean serum elimination half-lives similar to those calculated for males, respectively, for PFOS (5.9 years vs. 5.4 years; *p* = 0.87) and PFOA (3.3 years vs. 3.8 years; *p* = 0.69), and non-significantly higher for PFHS (12.8 years vs. 8.2 years; *p* = 0.23).

Positive Pearson correlation coefficients were observed for the log transformations of the half-life (days) of elimination for PFOS and initial (*r* = 0.36; *p* = 0.07) and end-of-study PFOS concentrations (*r* = 0.60; *p* = 0.001). For PFHS, the Pearson correlation coefficients were 0.27 (*p* = 0.18) and 0.46 (*p* = 0.02), respectively. Similarly for PFOA, the Pearson correlation coefficients were −0.04 (*p* = 0.86) and 0.31 (*p* = 0.12). Age at study onset, time worked, usual 3M job, and time since retirement were not associated with PFOS, PFHS, or PFOA serum elimination half-lives. A review of each subject’s self-reported diseases and medications did not provide additional evidence of possible associations with the half-lives of serum elimination. No renal diseases were reported by the 26 subjects. One individual reported an incidence of hepatitis during the 5-year study period.

## Discussion

Retirees were the population of choice for this study for three important reasons: *a*) to minimize the possibility of occupational exposure as compared to a working population potentially exposed to these perfluoroalkyl acids and their salts; *b*) to have serum concentrations higher than the general population in order to minimize any influence that nonoccupational sources of exposure might have on the determination of the serum elimination rate; and *c*) to have serum concentrations sufficiently measurable over time to mitigate any methodologic issues regarding evaluation of trend data in the presence of detection limits.

It is unlikely that the potential for non-occupational exposures substantially distorted the elimination rates calculated as median end-of-study concentrations for PFOS (295 ng/mL), PFHS (117 ng/mL), and PFOA (148 ng/mL), which remained above comparable estimates of 30.4, 2.1, and 5.2 ng/mL, respectively, reported for a statistically representative sample of the U.S. general population (Calafat et al. 2007). Furthermore, only four study subjects (subjects 1–4) had end-of-study PFOS concentrations that were below the upper 95% CI of the geometric mean of the 95th percentile as reported by Calafat et al. (97.5 ng/mL). Subject 1 had an end-of-study concentration at the upper 95% confidence limit of the geometric mean of the 95th percentile for PFHS (10 ng/mL), and no subject was below this upper bound for PFOA (13.5 ng/mL). It is possible that the rate of elimination may have resulted in more shallow slopes as concentrations declined because of the influence of environmental exposure.

The actual pharmacokinetics of PFOS, PFHS, and PFOA in humans is not likely to be consistent with a one-compartment distribution in spite of the present data fit to a first order model. Andersen et al. (2006) showed that intravenous dosing with PFOS or PFOA in cynomolgus monkeys produced time-course curves consistent with a two-compartment (tissue and renal filtrate) distribution. Nevertheless, the present study data strongly indicate that humans are very slow eliminators of these three perfluoroalkyl acids compared with other species.

The serum elimination half-life of PFOA in the present study’s 26 retired fluorochemical production workers is considerably longer than the elimination half-life in cynomolgus monkeys (approximately 14–40 days) after either repeated daily oral doses or a single intravenous injection (Butenhoff et al. 2004). Beagle dogs (Hanijärvi et al. 1988) and mice (Hundley et al. 2006) were found to have serum elimination half-lives of PFOA in the same range as monkeys. Both sexes of rabbits, however, excreted PFOA with elimination half-lives in hours (Hundley et al. 2006). In the rat, not only is the elimination half-life of PFOA shorter than that in monkeys and humans, but a notable sex difference exists (Kemper 2003; Kudo et al. 2002; Vanden Heuval et al. 1991). Whereas α−2u-globulin was ruled out as a cause for the slower elimination in male rats (Han et al. 2004), the marked difference between sexes in rats could be attributable to sex hormone regulation of the expression of certain organic anion transporters [OAT2 (Slc22a7), OAT3 (Slc22a8), and oatp1 (Slco1a1)] in the kidney (Kudo et al. 2002). Kudo et al. (2002) reported OAT2 (Slc22a7) to be more highly expressed in female rat kidney and subject to up-regulation by estradiol.

The diversity of proximal tubular organic anion transporters and potential for genetic variation (Eraly et al. 2004; Kudo et al. 2002; Ljubojevic et al. 2004) have indicated that the long elimination half-life in humans compared with that in other species may be due to differential expression of organic anion transporters and could be linked to either low-level transport into urine or increased tubular resorption. Harada et al. (2005) reported sex-independent renal clearances in humans that were 10^−5^-fold lower than the glomerular filtration rate indicating a significant absence of active renal excretion of PFOS and PFOA. Using a physiologically motivated pharmaco-kinetic model for renal clearance, Andersen et al. (2006) described the cynomolgus monkey pharmacokinetic data for PFOS (Noker and Gorman 2003a; Seacat et al. 2002) and PFOA (Butenhoff et al. 2002, 2004) in terms of renal resorption via high efficiency transporters. Although specific transporters were not identified, Andersen et al. (2006) concluded from their model simulations that saturable, high-affinity resorption processes govern the kinetics of PFOS and PFOA, and likely other perfluoroalkyl acids (e.g., PFHS), which could account for the varied half-lives of elimination across species. In their models, PFOS had a higher transport capacity and lower affinity than PFOA.

In the present study, the observation that linear PFOA is the predominant isomer in these retired workers may also indicate pharmacokinetic differences in either preferential lack of absorption and/or increased elimination of branched isomers of PFOA. This observation is supported by Loveless et al. (2006) who reported that branched APFO dosages administered orally to rats resulted in considerably lower serum PFOA concentrations compared with the same dosages (3, 10, and 30 mg/kg/day) of 100% linear or an 80% linear/20% branched mixture of APFO, although additional differences could also be attributable to variation in isomer absorption in the gastrointestinal tract. In mice, PFOA concentrations were similar regardless of the same three isomer types of APFO material and six dosages administered. Lau et al. (2005) reported that the terminal serum half-life of PFOA in mice was in the range of 15–20 days, with no sex-dependent differences in elimination.

It is also possible that differences in biliary excretion and gut resorption could account, in part, for the longer elimination half-life in humans because of the enterohepatic circulation observed (Johnson et al. 1984; Vanden Heuvel et al. 1991). However, in light of the recent evidence presented by Andersen et al. (2006), enterohepatic circulation is likely a lesser explanation.

Less is known about the elimination kinetics of PFHS in other species. The results indicate the human half-life of serum elimination may be longer and more variable than that of PFOS. A greater degree of variability has also been observed for PFHS compared with PFOS in intravenous dosing studies of cynomolgus monkeys (Noker and Gorman 2003a; 2003b). The half-life of serum elimination for PFHS ranged between 100 and 200 days (mean, 141 days) in three male monkeys and between 49 and 140 days (mean, 87 days) in three female monkeys (Noker and Gorman 2003b) compared with a more narrow range for PFOS of 122–146 days (mean, 132 days) and 88–138 days (mean, 110 days) for male and female monkeys, respectively (Noker and Gorman 2003a).

## Figures and Tables

**Table 1:** Demographic characteristics of study participants by subject number.

Subject no.	Sex	Facility	Usual job category	No. years worked	Years retired at initial collection	Age at initial collection (years)	Days followed	Years followed	Samples analyzed
1	Male	Decatur	Laboratory/technician	32	0.6	59	1,945	5.3	8
2	Male	Decatur	Supervisor	30	2.1	62	551 or 1,945^a^	2.2 or 5.3^a^	4 or 8^a^
3	Male	Decatur	Supervisor	27	3.1	65	1,730 or 1,945^b^	4.7 or 5.3^b^	7 or 8^b^
4	Male	Decatur	Maintenance	33	3.7	61	1,945	5.3	8
5	Male	Cottage Grove	Chemical operator	28	11.5	75	1,730	4.7	7
6	Male	Decatur	Cell operator	29	1.9	63	1,945	5.3	8
7	Female	Decatur	Chemical operator	20	2.5	65	1,945	5.3	8
8	Male	Decatur	Maintenance	29	3.5	59	1,945	5.3	8
9	Male	Decatur	Foreman	36	1.9	60	1,139^c^	3.1	4
10	Male	Decatur	General worker	35	1.9	57	1,945	5.3	8
11	Male	Decatur	Maintenance	27	0.9	64	1,945	5.3	8
12	Male	Cottage Grove	Foreman	36	7.5	64	1,730	4.7	7
13	Male	Decatur	Laboratory/technician	36	1.9	59	1,945	5.3	8
14	Male	Decatur	Laboratory/technician	32	0.9	59	1,945	5.3	8
15	Male	Decatur	Cell operator	30	2.9	63	1,945	5.3	8
16	Male	Decatur	Foreman	35	1.9	58	1,945	5.3	8
17	Male	Decatur	Maintenance	33	1.9	59	1,945	5.3	8
18	Male	Decatur	Maintenance	21	1.0	63	1,524^d^	4.2	6
19	Male	Decatur	Laboratory/technician	33	1.1	59	1,945	5.3	8
20	Male	Decatur	Foreman	34	1.5	58	1,524^d^	4.2	7
21	Male	Decatur	General worker	30	0.9	56	1,730^e^	4.7	7
22	Male	Decatur	Cell operator	33	1.2	58	1,945	5.3	8
23	Male	Decatur	Foreman	34	3.1	59	1,945	5.3	8
24	Male	Cottage Grove	Chemical operator	36	7.5	65	1,744	4.8	7
25	Female	Decatur	Chemical operator	22	0.4	55	1,945	5.3	8
26	Male	Decatur	Chemical operator	31	0.9	61	1,945	5.3	8

a PFHS samples were < LOQ after 551 days; therefore, analyses included only 4 samples (> LOQ). All PFOS and PFOA samples were > LOQ; therefore, analyses included 8 samples collected through 1,945 days.

b Initial sample was analyzed only for PFOA because insufficient sample remained to analyze for all three compounds due to prior interim analyses. Therefore, follow-up was 1,730 days for PFOS and PFHS (7 samples) and 1,945 days for PFOA (8 samples).

c Subject had potential for occupational exposure through day 806 in study; therefore, we included only the last 1,139 days of follow-up, with 4 samples analyzed.

d Subject died during study collection period; the 1,524 days follow-up included 6 samples analyzed for subject 18, and 7 samples analyzed for subject 20.

e Subject had potential for occupational exposure through day 215 of study; therefore, follow-up included last 1,730 days, with 7 samples analyzed.

**Table 2:** Initial and final concentration and half-life of elimination in serum for PFOS, PFHS, and PFOA (ng/mL) by subject number.

	PFOS					PFHS					PFOA				
			Half-life					Half-life					Half-life		
Subject no.	Initial conc.	Final conc.	Days	SE	Years	Initial conc.	Final conc.	Days	SE	Years	Initial conc.	Final conc.	Days	SE	Years
1	145	70	1,598	203	4.4	63	27	1,440	336	3.9	74	26	1,297	86	3.6
2	156	37	885	38	2.4	16	10	798	208	2.2	72	17	830	67	2.3
3	218	82	1,411	152	3.9	58	29	4,373	4,387	12.0	490	129	1,031	63	2.8
4	258	74	1,124	65	3.0	86	37	1,971	436	5.4	142	51	1,314	151	3.6
5	259	148	2,304	265	6.3	157	110	3,508	789	9.6	1,077	404	1,205	66	3.3
6	323	129	1,273	141	3.5	647	424	2,284	463	6.3	430	108	858	49	2.3
7	443	242	1,792	203	4.9	114	86	4,458	1,599	12.2	1,622	577	1,221	80	3.3
8	477	187	1,436	57	3.9	193	113	2,347	245	6.4	306	188	2,518	195	6.9
9	545	424	2,761	749	7.6	54	48	5,329	2,978	14.6	254	150	1,401	168	3.8
10	551	207	1,387	65	3.8	191	96	1,923	186	5.3	131	45	1,107	107	3.0
11	572	296	1,961	121	5.4	60	27	1,878	260	5.1	247	104	1,551	87	4.2
12	617	226	1,163	64	3.2	302	160	1,892	253	5.2	1,180	145	561	37	1.5
13	620	249	1,354	76	3.7	191	121	3,028	370	8.3	181	65	1,280	71	3.5
14	632	213	1,224	99	3.4	149	44	1,065	145	2.9	183	50	1,020	81	2.8
15	691	391	2,456	296	6.7	494	365	4,000	934	11.0	356	244	3,334	641	9.1
16	766	383	1,871	125	5.1	373	195	2,266	170	6.2	167	78	1,737	241	4.8
17	846	430	2,464	526	6.7	273	161	3,836	1,708	10.5	212	84	1,385	142	3.8
18	924	293	908	110	2.5	269	99	1,024	1,428	2.8	390	61	570	48	1.6
19	929	802	7,919	3,126	21.7	131	102	4,939	2,036	13.5	496	284	2,552	214	7.0
20	1,033	500	1,484	56	4.1	398	256	2,825	1,173	7.7	702	248	1,041	72	2.9
21	1,079	500	1,737	349	4.8	252	144	2,134	595	5.8	549	235	1,479	208	4.0
22	1,090	478	1,566	85	4.3	928	610	3,637	776	10.0	474	162	1,235	29	3.4
23	1,190	539	1,723	137	4.7	293	154	1,983	108	5.4	425	162	1,358	69	3.7
24	1,420	1,003	3,122	287	8.5	364	361	9,858	6,962	27.0	5,100	2,435	1,662	69	4.6
25	1,500	839	2,475	225	6.8	193	164	4,866	1,428	13.3	883	266	1,223	107	3.3
26	3,490	1,740	1,973	114	5.4	1,295	791	3,180	680	8.7	1,833	486	1,061	72	2.9

conc., concentration.

**Table 3:** Measures of central tendency for half-life of serum elimination for PFOS, PFHS, and PFOA for 26 retired fluorochemical production workers.

	Days				Years			
Fluorochemical	Arithmetic mean (95% CI)	GM (95% CI)	Median	Range	Arithmetic mean (95% CI)	GM (95% CI)	Median	Range
PFOS	1,976^a^ (1,434–2,517)	1,751 (1,461–2,099)	1,661	885–7,919	5.4^a^ (3.9–6.9)	4.8 (4.0–5.8)	4.6	2.4–21.7
PFHS	3,109^b^ (2,348–3,870)	2,662 (2,112–3,355)	2,586	798–9,858	8.5^b^ (6.4–10.6)	7.3 (5.8–9.2)	7.1	2.2–27.0
PFOA	1,378 (1,131–1,625)	1,273 (1,083–1,495)	1,257	561–3,334	3.8 (3.1–4.4)	3.5 (3.0–4.1)	3.4	1.5–9.1

GM, geometric mean.

a If subject 19 is excluded as a potential high outlier for PFOS (Figure 2), the arithmetic mean half-life = 1,738 days (95% CI, 1,497–1,979) or 4.8 years (95% CI, 4.1–5.4).

b If subject 24 is excluded as potential high outlier for PFHS (Figure 2), the arithmetic mean half-life = 2,839 days (95% CI, 2,297–3,381) or 7.8 years (95% CI, 6.3–9.3).

## References

[b1-ehp0115-001298] Andersen ME, Clewell HJ, Tan YM, Butenhoff JL, Olsen GW (2006). Pharmacokinetic modeling of saturable, renal resorption of perfluoroalkylacids in monkeys–probing the determinants of long plasma half-lives. Toxicology.

[b2-ehp0115-001298] Butenhoff J, Costa G, Elcombe C, Farrar D, Hansen K, Iwai H (2002). Toxicity of ammonium perfluorooctanoate (APFO) in male cynomolgus monkeys after oral dosing for six months. Toxicol Sci.

[b3-ehp0115-001298] Butenhoff JL, Kennedy GL, Hinderliter PM, Lieder PH, Jung R, Hansen KJ (2004). Pharmacokinetics of perfluoro-octanoate (PFOA) in cynomolgus monkeys. Toxicol Sci.

[b4-ehp0115-001298] Butenhoff JL, Olsen GW, Pfahles-Hutchens A (2006). The applicability of biomonitoring data for perfluorooctanesulfonate (PFOS) to the environmental public health continuum. Environ Health Perspect.

[b5-ehp0115-001298] Calafat AM, Kuklenyik Z, Reidy JA, Caudill SP, Tully JS, Needham LL (2007). Serum concentrations of 11 polyfluoro-alkyl compounds in the U.S. population: data from the National Health and Nutrition Examination Survey (NHANES) 1999–2000. Environ Sci Technol.

[b6-ehp0115-001298] Eraly SA, Bush KT, Sampogna RV, Bhatnagar V, Nigam S (2004). The molecular pharmacology of organic anion transporters: from DNA to FDA?. Mol Pharmacol.

[b7-ehp0115-001298] Han X, Hinderliter PM, Snow TA, Jepson GW (2004). Binding of perfluorooctanoic acid to rat liver-form and kidney-form α2u-globulins. Drug Chem Toxicol.

[b8-ehp0115-001298] Hanijärvi H, Beynen AC, Solleveld HA (1988). A proposed species difference in the renal excretion of perfluorooctanoic acid in the beagle dog and rat. New Developments in Biosciences: Their Implications for Laboratory Animal Science.

[b9-ehp0115-001298] Harada K, Inoue K, Morkawa A, Yoshinaga T, Saito N, Koizumi A (2005). Renal clearance of perfluorooctane sulfonate and perfluorooctanoate in humans and their species-specific excretion. Environ Res.

[b10-ehp0115-001298] Houde M, Martin JW, Letcher RJ, Solomon KR, Muir DCG (2006). Biological monitoring of polyfluoroalkyl substances: a review. Environ Sci Technol.

[b11-ehp0115-001298] Hundley SG, Sarrif AM, Kennedy GL (2006). Absorption, distribution, and excretion of ammonium perfluorooctanoate (APFO) after oral administration to various species. Drug Chem Toxicol.

[b12-ehp0115-001298] Johnson JD, Gibson SJ, Ober RE (1979). Extent and Route of Excretion and Tissue Distribution of Total Carbon-14 in Rats after a Single i.v. Dose of FC-95-^14^C. U.S. EPA docket AR-226–0006.

[b13-ehp0115-001298] Johnson JD, Gibson SJ, Ober RE (1984). Cholestyramine-enhanced fecal elimination of carbon-14 in rats after administration of ammonium [^14^C]perfluorooctanoate or potassium [^14^C]perfluorooctanesulfonate. Fundam Appl Toxicol.

[b14-ehp0115-001298] Kemper RA (2003). Perfluorooctanoic Acid: Toxicokinetics in the Rat. Laboratory Project ID:DuPont-7473. U.S. EPA docket AR-226–1350.

[b15-ehp0115-001298] Kerstner-Wood C, Coward L, Gorman G (2003). Protein Binding of Perfluorobutane Sulfonate, Perfluorohexane Sulfonate, Perfluorooctane Sulfonate and Perfluorooctanoate to Plasma (Human, Rat and Monkey), and Various Human-Derived Plasma Protein Fractions. U.S. EPA docket AR-226–1354.

[b16-ehp0115-001298] Kudo N, Katakura M, Sato Y, Kawashima Y (2002). Sex hormone-regulated renal transport of perfluorooctanoic acid. Chem Biol Interact.

[b17-ehp0115-001298] Kudo N, Kawashima Y (2003). Toxicity and toxicokinetics of perfluorooctanoic acid in humans and animals. J Toxicol Sci.

[b18-ehp0115-001298] Kuslikis BI, Vanden Heuvel JP, Peterson RE (1992). Lack of evidence for perfluorodecanoyl- or perfluorooctanoyl-coenzyme A formation in male and female rats. J Biochem Toxicol.

[b19-ehp0115-001298] Lau C, Strynar MJ, Lindstrom AB, Hanson RG, Thibodeaux JR, Barton HA (2005). Pharmacokinetic evaluation of perfluorooctanoic acid in the mouse [Abstract]. Toxicologist.

[b20-ehp0115-001298] Ljubojevic M, Herak-Kramberger CM, Hagos Y, Bahn A, Endou H, Burckhardt G (2004). Rat renal cortical OAT1 and OAT3 exhibit gender differences determined by both androgen stimulation and estrogen inhibition. Am J Physiol Renal Physiol.

[b21-ehp0115-001298] Loveless SE, Finlay C, Everds NE, Frame SR, Gillies PJ, O’Connor JC (2006). Comparative responses of rats and mice exposed to linear/branched, linear, or branched ammonium perfluorooctanoate (APFO). Toxicology.

[b22-ehp0115-001298] Luebker DJ, Hansen KJ, Bass NM, Butenhoff JL, Seacat AM (2002). Interactions of fluorochemicals with rat liver fatty acid-binding protein. Toxicology.

[b23-ehp0115-001298] Medinsky MA, Klaassen CD, Klaassen CD, Amdur MO, Doull J (1996). Toxicokinetics. Casarett & Doull’s Toxicology.

[b24-ehp0115-001298] Noker PE, Gorman GS (2003a). A pharmacokinetic study of potassium perfluorooctanesulfonate in the cynomolgus monkey. U.S. EPA docket AR-226–1356.

[b25-ehp0115-001298] Noker PE, Gorman GS (2003b). A pharmacokinetic study of potassium perfluorohexanesulfonate in the cynomolgus monkey. U.S. EPA docket AR-226–1361.

[b26-ehp0115-001298] Seacat AM, Thomford PJ, Hansen KJ, Olsen GW, Case MT, Butenhoff JL (2002). Subchronic toxicity studies on perfluorooctanesulfonate potassium salt in cynomolgus monkeys. Toxicol Sci.

[b27-ehp0115-001298] Vanden Heuvel J, Kuslikis B, Van Refelghem M, Peterson R (1991). Tissue distribution, metabolism and elimination of perfluorooctanoic acid. J Biochem Toxicol.

